# Critical physiological and pathological functions of Forkhead Box O tumor suppressors

**DOI:** 10.15190/d.2013.5

**Published:** 2013-12-31

**Authors:** Georgiana R. Dumitrascu, Octavian Bucur

**Affiliations:** "Victor Babes" National Institute of Pathology and Biomedical Sciences, Bucharest, Romania; Department of Pathology, Harvard Medical School and Beth Israel Deaconess Medical Center, Boston, MA, USA

**Keywords:** FOXO, FOX, FOXO3, FOXO1, apoptosis, cell cycle, metabolism, cancer, stem cells, cancer stem cells, differentiation, microRNAs, small non-coding RNAs

## Abstract

The Forkhead box, subclass O (FOXO) proteins are critical transcription factors, ubiquitously expressed in the human body. These proteins are characterized by a remarkable functional diversity, being involved in cell cycle arrest, apoptosis, oxidative detoxification, DNA damage repair, stem cell maintenance, cell differentiation, cell metabolism, angiogenesis, cardiac development, aging and others. In addition, FOXO have critical implications in both normal and cancer stem cell biology. New strategies to modulate FOXO expression and activity may now be developed since the discovery of novel FOXO regulators and non-coding RNAs (such as microRNAs) targeting FOXO transcription factors. This review focuses on physiological and pathological functions of FOXO proteins and on their action as fine regulators of cell fate and context-dependent cell decisions. A better understanding of the structure and critical functions of FOXO transcription factors and tumor suppressors may contribute to the development of novel therapies for cancer and other diseases.

## SUMMARY

1. Introduction
2. Structure of FOXO family members
3. Physiological functions of FOXO proteins
4. Implications of FOXO proteins in pathological processes and associated diseases
5. Critical functions of FOXO transcription factors in stem cells and their role in cancer stem cells
6. Expression of FOXO family members in various tissues
7. FOXOs and microRNAs
8. Conclusions and future perspectives

## 1. Introduction

The Forkhead box (FOX) proteins represent a wide family of transcription factors that display an extraordinary functional diversity, regulating a variety of critical biological processes^1,2^. FOX proteins are well known to control the following physiological procceses: apoptosis, cell-cycle, cellular metabolism, immune response, differentiation, development (such as cardiac development) or aging. FOX proteins are also involved in various pathologies, such as cancer, diabetes and neurodegenerative diseases^3,4^.

The first forkhead transcription factor was first identified in 1989, and its function was related to the development of the anterior and posterior gut of the Drosophila embryo. The “forkhead” name was given because of the two spiked-head structures found in the embryos of the *Drosophila Melanogaster forkhead* mutant presenting modifications of the gut formation^5^. One year later, the sequence comparison between forkhead and mammalian hepatocyte-enriched transcription factor HNF-3A showed a conserved 110-amino-acid DNA binding domain, bringing evidence that forkhead proteins are a new class of transcription factors^6^.

Hence, forkhead transcription factors are defined by their winged-helix DNA binding domain, a conserved structure called „Forkhead box”, the symbol FOX being assigned to all vertebrate forkhead transcription factors, according to the revised nomenclature^7^.

Since its discovery, numerous forkhead genes have been identified in a broad range of organisms, from yeast and worms to humans^2^. Interestingly, up to now, FOX genes have not been identified in plants^8^. However, an update published in 2010 reported that are 50 FOX genes identified and classified in the human genome and 44 in the mouse genome^9^. Therefore, a standard nomenclature system was required for this extended number of discovered factors. In 2000, Daniel E. Martinez and his team established Fox Nomenclature Committee, the first step towards an unified nomenclature for the winged-helix / forkhead transcription factors. The Committee has changed the initial terms for the forkhead proteins (e.g. FREAC - Forkhead RElated Activator; fkh - Drosophila gene fork head)^7^. Currently, based on phylogenetic analysis, FOXO genes are classified in subclasses that range from FOXA to FOXS to yield 23 subclasses in total^10-13^. Each subclass has several members noted with an arabic number. While for human forkhead proteins abbreviations have all letters in uppercase (e.g., FOXD3), for mouse only the first letter is capitalized (e.g., Foxd3) and for all other chordates the first and subclass letter are in uppercase (e.g., FoxD3)^7^.

One of the largest and the most important subclass of FOX family is represented by FOXO (Forkhead box, subclass O) transcription factors. FOXO transcription factors (FOXOs) are characterized by the same conserved DNA binding domain that define the family of forkhead proteins^1^. However, FOXOs share an additional unique 5 amino acid sequence insertion within the DNA binding domain that is not present in other forkhead proteins^14^.

Only one FOXO transcription factor is known in invertebrates (named abnormal dauer formation protein 16/DAF-16 in the nematode worm Caenorhabditis elegans and dFOXO in the fruit fly Drosophila melanogaster), while in mammals four FOXO proteins encoded by four different genes were identified: FOXO1, FOXO3, FOXO4 and FOXO6^15^.

Initially, FOXO1 transcription factor (previously known as FKHR-forkhead in rhabdomyosarcoma) was identified through its involvement in chromosomal translocations t(2;13) and t(1;13) in alveolar rhabdomyosarcoma tumors due to PAX3/7-FKHR fusion transcript^16,17^. A few years later, FOXO3 (previously known as FKHRL1 - forkhead in rhabdomyosarcoma like protein 1) was characterized and named, based on similarities to FKHR^18^. The gene for FOXO4 was described as being fused to MLL transcription factor as a result of the t(X; 11) chromosomal translocation in acute lymphoblastic leukemia, therefore, the initial term for FOXO4 was AFX (The acute leukemia fusion gene located on chromosome X)^19^.

FOXO proteins are considered unique cellular targets, regulating a wide variety of critical cellular processes, such as: apoptosis, oxidative stress, DNA damage repair, cell cycle, stem cell proliferation and maintenance, metabolism, angiogenesis, vascular tone, cardiovascular development, fertility, immune response and neuronal survival^4,20^. The aim of this review is to discuss the most recent advances in elucidating the functions, structure and transcriptional regulation of Forkhead Box O transcription factors, both in physiological and pathological conditions. The advances in understating the mechanism of FOXOs regulation of stem cells, cancer stem cells and how non-coding RNAs are regulated and regulate the function of FOXO genes/protein are presented.

## 2. Structure of FOXO family members

**Figure 1:**
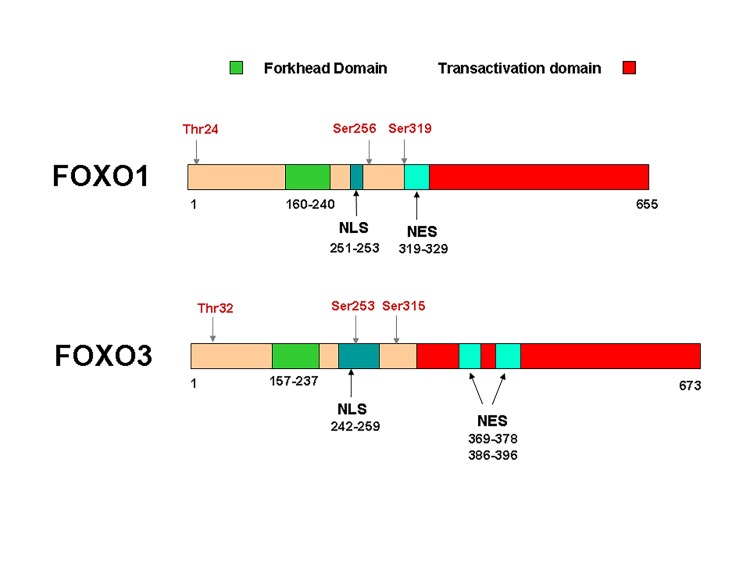
Structure of FOXO family members FOXO1 and FOXO3 are exemplified here. In humans, the primary structure of the FOXO proteins is characterized by a length of approximately 655-675 amino acid residues (aa) for FOXO1 (655 aa) and FOXO3 (673 aa), while FOXO4 has 505 aa and FOXO6 492 aa All members of the FOXO family consist of four domains: a forkhead DNA-binding domain (DBD), a nuclear localization signal (NLS), a nuclear export sequence (NES) and a C-terminal transactivation domain.

In humans, the primary structure of the FOXO proteins is characterized by a length of approximately 655-675 amino acid residues (aa) for FOXO1 (655 aa) and FOXO3 (673 aa), and a shorter sequence of approximately 500 amino acids for FOXO4 (505 aa) and FOXO6 (492 aa)^21-24^. All members of the FOXO family consist of four domains: a forkhead DNA-binding domain (DBD), a nuclear localization signal (NLS), a nuclear export sequence (NES) and a C-terminal transactivation domain^25^ (**Figure 1**).

The forkhead DNA-binding domain (DBD), also called the forkhead box, is described as a „winged helix” due to the butterfly-like aspect on X-ray crystallography and nuclear magnetic resonance^26^. Analysis of the amino acid sequences alignment of FOXO proteins revealed a highly conserved DNA binding domain among all the members of the broader group of forkhead transcription factors, but also among species, in eukaryotic organisms, from yeast to humans^3,27^.Moreover, several studies point out that forkhead DBD is not the only region in the FOXO molecule that is highly conserved. Similarities were also observed in the N-terminal region near the first AKT/protein kinase B (PKB) phosphorylation site (Thr24 for FOXO1 and Thr32 for FOXO3), the region containing NLS, and sequences from C-terminal transactivation domain^25^.

As mentioned above, the forkhead domains in FOXO subclass of FOX family**are similar to the forkhead regions of other subclasses*. *For example, structural similarities have been proven in the core region of FOXO3 compared to FOXA3, FOXK1 and FOXP2^28^. The forkhead/winged helix motif is a shared sequence between subclasses of FOXO, of around 100 amino acids that folds into three alpha-helices (H1, H2 and H3), three beta-strands (S1, S2 and S3) and two wing-like loops (W1 and W2). The structure has a H1 - S1 - H2 - H3 - S2 - W1 - S3 - W2 topology and the strands S1, S2 and S3 interact with each other forming a beta-sheet. While the N-terminal part of the domain is formed by a cluster of three alpha-helices, the C-terminal half consists of beta strands S2 and S3 and two large loops (wings W1 and W2)^25^. The main DNA recognition site is represented by the highly conserved helix H3 of forkhead DNA-binding domain. The stability of forkhead DBD - DNA complexes is increased by the more variable regions of the DBD, including H1, the region between H2 and H3, wing W1 and C-terminal segment^25^.

Although the FOXO forkhead box is clearly related to those found in other forkhead genes, all FOXO members contain an aditionally segment of five amino acid (198-GDSNS-202) between helices H2 and H3, that creates a small extra loop, forming a coil structure in the FOXO3 DBD, and a short helix in the FOXO4 DBD^28,29^.

This is important in sequence-specific interactions with DNA-binding sites^30^.

FOXO proteins mediate transcriptional activation through binding to the conserved consensus core motif TTGTTTAC in the DNA^31^. They bind DNA through a FOXO-recognized element with a T/C-G/A-A-A-A-C-A-A consensus sequence in the forkhead DBD (C-terminal)^26^. There are fourteen protein-DNA contacts described, which mediate the activation/inhibition of FOXO’s target genes, such as Bim, TRAIL, p27, p21 and catalase. The main recognition site is the α-helix H3^32^.

## 3. Physiological functions of FOXO proteins

Forkhead proteins function as transcription factors that bind to DNA through their forkhead domain in order to upregulate or downregulate the expression of a tremendous number of target genes^20^.

**Figure 2:**
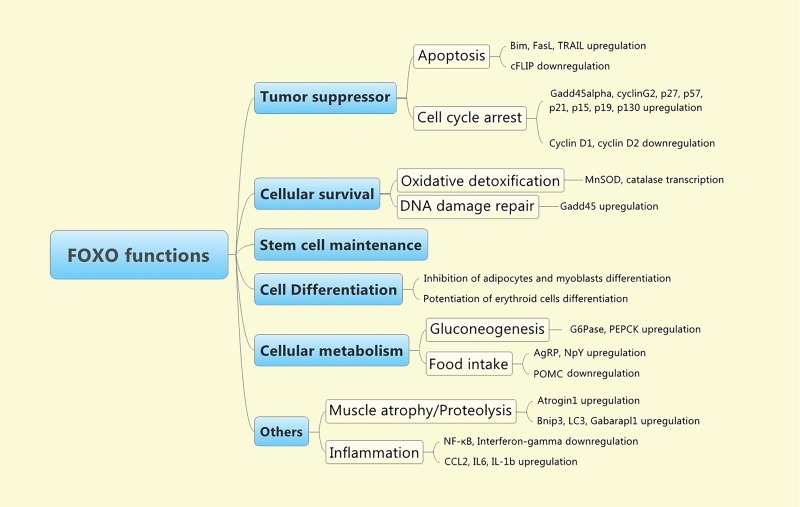
Functions of FOXO family of transcription factors FOXO transcription factors control the expression of a wide spectrum of genes that regulate essential physiological cellular processes, such as cell death/cell survival, cell cycle, cell proliferation, cell differentiation/development, angiogenesis, cell metabolism, stress response and stem cell maintenance.

Hence, the FOXO transcription factors control the expression of a wide spectrum of genes that regulate essential physiological cellular processes, such as cell death/cell survival, cell cycle, cell proliferation, cell differentiation/development, angiogenesis, cell metabolism, stress response and stem cell maintenance^33-38^ (**Figure 2**). Moreover, FOXOs are also involved in a wide range of pathological cellular processes, such as cancer, neurodegenerative diseases (Parkinson’s disease, Alzheimer), diabetus mellitus, cardiac failure, atherosclerosis, hypertension and anovulation^39-46^.

Differences between functions and regulatory mechanisms of FOXO1 and FOXO3 proteins are considered to be partially redundant, with several exceptions. For example, AKT phosphorylates all FOXO family members, inducing their translocation into the cytoplasm and inactivation, while PP2A dephosphorylates part of the AKT phosphorylation sites in FOXO1 and FOXO3, reactivating them^47^. Several particular differences regarding the regulatory kinases, E3 ligases or other important enzymes and regulators exist. Thus, their activity is in part controlled by different mechanisms^31^.

### Apoptosis

FOXO transcription factors regulate the expression of multiple pro-apoptotic and anti-apoptotic proteins, and they have the ability to induce apoptosis by activating either intrinsic or extrinsic pathways of apoptosi^48-52^. Moreover, the consensus FOXO recognition element (FRE) - (G/C)(T/A)AA(C/T)AA - which differs from that of other forkhead proteins, seems to have a very important role in both apoptotic pathways, since matching functional FRE sites have been identified in the promoters of FOXO target genes encoding Fas ligand (FasL), insulin like growth factor-binding protein 1 (IGFBP1), the apoptotic regulator Bcl-2 interacting mediator of cell death (Bim) and others^30^.

FOXO triggers the mitochondria-dependent intrinsec apoptotic pathway through upregulation of multiple pro-apoptotic Bcl-2 family members, such as Puma, Bim, NOXA, BNIP3, and downregulation of the anti-apoptotic Bcl-2 family member Bcl-xL^48-52^. The expression of the anti-apoptotic protein Bcl-xL is suppressed by FOXO proteins, such as FOXO4, after increasing the expression of the transcriptional repressor Bcl-6, in order to trigger apoptosis^52^.

Both FOXO-induced expression of the pro-apoptotic Bcl-2 family members and FOXO-downmodulated anti-apoptotic Bcl-2 family members lead to apoptosis due to mitochondrial outer membrane permeabilization (MOMP), as a response to intracellular stress, growth factor deprivation or other factors^53^. These mitochondrial modifications result in release of cytochrome c, Smac/DIABLO and Omi/HtrA2 from mitochondria, subsequently triggerring down-stream caspases activation^54,55^. Apaf 1 binds cytochrome c forming a complex named Apoptosome, required for caspase 9 activation (initiator caspase)^54^. Caspase 9 activation further activates the effector caspases, such 3, 6 or 7, triggering caspase cascade^54^.

FOXO proteins are also able to induce apoptosis by activating the receptor-dependent extrinsic pathway of apoptosis. They induce the upregulation of the death receptor ligands FasL and TRAIL, promoting an autocrine and/or paracrine action of these death ligands on the death receptors^56^. Fas signaling pathway is important in apoptosis induction through the extrinsic pathway, since Jurkat cells lacking several critical components of Fas pathway fail to induce FOXO-dependent cell death^57^. Upregulation of tumor necrosis factor related apoptosis inducing ligand (TRAIL) by increased levels of FOXO1 or FOXO3 in cancer cells, such as prostate carcinoma cells, leads to apoptosis^58^. Thus, binding of Fas ligand (FasL) and TRAIL to their receptors (Fas/CD95/APO-1 for FasL and DR4, DR5 for TRAIL) triggers a death-inducing signaling complex (DISC) with a subsequent activation of caspases, mainly initiator caspase 8 and effector caspase 3, leading to apoptosis^59^.**Moreover, FOXO transcription factors directly regulate the expression of tumor necrosis factor receptor-associated death domain (TRADD). Activation of FOXO proteins by PI3K-AKT pathway inhibitors results in increased expression of TRADD protein^60^. TRADD is an important adaptor protein interacting with TNFR1 and Fas receptors, mediating apoptosis and NFkB pathway activation^61^.

### Cell cycle and proliferation

Under normal physiological conditions,**cyclins and their associate cyclin dependent kinases (CDKs) are very important for the progression of the cell cycle^62^. In response to DNA damage, FOXO transcription factors increase the expression levels of the CDK inhibitors binding to cyclin/CDK complexes, such as p21 (also known as CDK inhibitor 1) and p27 (Kip1)^63,64^. CDK inhibitors together with FOXO-induced inhibition of Cyclins expression act by stopping the cell cycle at different checkpoints in order to repair the DNA damage or to remove the damaged cell^65^. For example, a study performed on 32D murine myeloid cells and on BaF3 murine pre–B-cell lines brings evidence that endogenous FOXO proteins are required to enforce cell cycle checkpoints after DNA damage^33^.

The cell cycle arrest at G1/S transition is induced by FOXO by upregulating the negative regulators of the G1/S phase, such as the CDK inhibitors: p27^KIP1^, p57^KIP2^, p21^CIP1/WAF1 ^(CIP/KIP family), p15^INK^, p19^INK^ (INK family) and the retinoblastoma protein family member p130^27^.Also, FOXO decreases the positive regulators, such as cyclin D1, or cyclin D2, blocking the G1/S transition^66^. Additionally, FOXO increases the expression of negative regulators such as Gadd45alpha and cyclin G2, resulting in cell cycle arrest at G2/M^66^. Surprisingly, FOXO proteins act as transcription factors for PLK1 expression during cell cycle, PLK1 being critical in promoting G2-M phase transition, M phase progression and end of mitosis^67^. A complete knock-down of FOXO protein levels induces an arrest in cell cycle, suggesting that a basal, low levels of FOXOs activity is required for the cell cycle progression^67^. Surprisingly, in specific settings, FOXO may actually serve as a promoter of proliferation. For example, neutrophils isolated from FOXO3 deficient mice show high levels of FasL expression and apoptosis, revealing that FOXO3 may represses FasL expression in neutrophils, leading to proliferation in this type of cells^68^. FOXO1 was also shown to positively influence proliferation induction in pancreatic β cells in vitro, under low nutritional circumstances. The mechanisms implicated in this process are not completely understood. However, the induction of Ccnd1 gene transcription, which encodes Cyclin D1, may at least partially be involved. Cyclin D1 represents one of the earliest cell cycle-related events, being critical for the G1 to S phase progression during the cell cycle^34^.

Thus, FOXO proteins coordinate the expression of multiple important cell cycle regulators, in order to block the G0/G1, G1/S or the G2/M transitions during cell cycle when needed, such as after DNA damage. However, a basal level of FOXO proteins is required for G2-M cell cycle transition, when FOXO-dependent PLK1 expression seems to be necessary. These results suggest that FOXO proteins have to be tightly regulated and are important and sensitive regulators of cell cycle, proliferation and other critical cellular processes.**

### Differentiation and development

FOXO proteins play an important role in regulating differentiation of a wide variety of tissues. It is well known that FOXOs can control the differentiation of precursor cells into muscle, adipose tissue or blood cells. Interestingly, FOXOs effect on differentiation is context dependent and sometimes FOXO isoform dependent. While FOXO3 promotes differentiation of erythroid cells, FOXO1 suppresses the differentiation of precursor cells in adipose and muscle tissues^69^. FOXOs can also suppress bone formation by inhibiting Wnt-β catenin-TCF signaling. Wnt signaling is known to stimulate bone formation^70^.

Moreover, FOXO proteins are required for maintenance of somatic/adult stem cells, such as hematopoietic stem cells, and they also regulate cancer stem cells^71^ (see details in Chapter 5).

### Cellular Metabolism

Metabolic signaling mediated by forkhead transcription factors is conserved among multiple species, including but no limited to mammals, Drosophila melanogaster and Caenorhabditis elegans^20^. It was first noticed for the FOXO homolog named Dauer Formation-16 (DAF-16), in the Caenorhabditis elegans worm^72^.

FOXO proteins are involved in critical physiological processes that regulate cellular metabolic activity in many organs, such as liver, pancreas, adipose tissue and hypothalamus^73-76^. In the liver, FOXO1 forms a complex with another transcription factor, the liver specific PGC1alpha, in order to induce gluconeogenesis by upregulating G6Pase and PEPCK genes. This is important for maintaining glucose homeostasis^73^. Confirming these results, loss of hepatic FOXO genes in mice induces a downmodulation of gluconeogenesis and an upregulation of glycolysis^77^. In addition, ectopic expression of FOXO1 in rat primary hepatocytes increases apocIII, an inhibitor of lipoprotein lipase, suggesting that FOXO1 is involved in regulating the lipid metabolism^78^. Recently, nicotinamide phosphoribosyltransferase (Nampt) gene was described to be a new transcriptional target gene of FOXOs for regulating hepatic triglyceride levels^79^. In pancreas, FOXO1 plays an important role in the maintenance of beta cell function, but also in the development of the pancreas, through repression of the pancreatic transcription factor Pdx1^74^. Aditionally, FOXO1 increases the food intake by upregulating AgRP and NpY, and by downregulating POMC in hypothalamus, antagonizing the anorexigenic hormone leptin^76,80^. Recently, G-protein-coupled receptor Gpr17 was described to be a FOXO1 target that activates AgRP, suggesting a new mechanism for FOXO1-AgRP mediated food intake that might provide a new treatment for obesity^81^.

All these results establish FOXO proteins as important regulators of cellular metabolism and potential target in metabolic related diseases.

### Stress Response, DNA Damage Repair and Longevity

**Figure 3:**
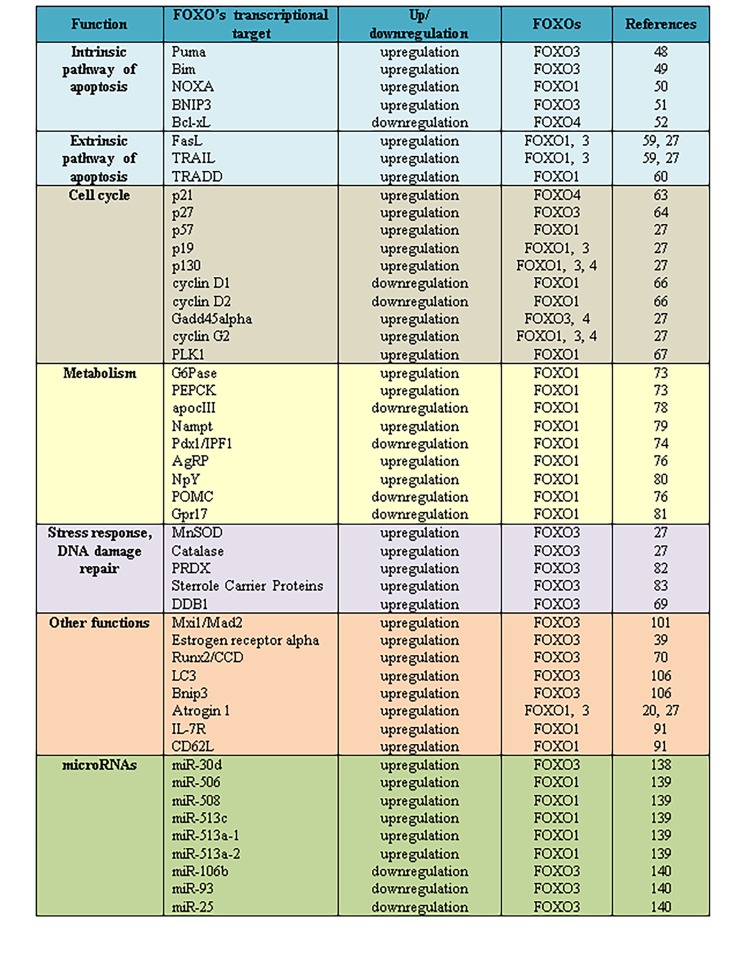
Selective list of FOXO regulated transcriptional targets

FOXO transcription factors function as sensors for reactive oxygen species (ROS) and play an important role in cellular resistance to stress, increasing cellular survival. FOXO proteins are able to protect the cell from oxidative damage, decreasing the availability of ROS. They stimulate the expression of certain genes responsible for the ROS inactivation, such as the antioxidant enzymes manganese superoxide dismutase (MnSOD), that catalyzes the conversion of superoxide to hydrogen peroxide, and catalase, that converts hydrogen peroxide into water and oxygen^27^. Studies on human cardiac fibroblasts revealed that FOXO also modulate the antioxidant enzyme peroxiredoxin III, fighting against cell damage induced by oxidative stress.**Concomitant peroxiredoxin III knockdown and FOXO3 knockdown resulted in higher levels of hydrogen peroxide in response to serum starvation, as compared to peroxiredoxin III knockdown only^82^. Other FOXO transcriptional targets (**Figure 3**), such as sterrole carrier proteins (SCPs), are now known to play an important role during the defence against oxidative stress. SCPs are implicated in protecting fatty acids from peroxidation^83^. FOXO proteins are also able to increase DNA repair, inducing a cell cylce arrest at G2/M checkpoint, in order to provide time for repairing the DNA damage. One of the transcriptional target of FOXO, implicated not only in cell cycle arrest but also in DNA repair and cell survival in response to cellular stress, is Gadd45^84^. Similarly, DDB1 gene protein product is able to repair the DNA damage through upregulation by FOXO^69^.

FOXOs regulate the longevity of the cell mainly by increasing resistance to stress, modulating the DNA repair process and maintaining the stem cells^27^. The lifespan extension is an important function of FOXO that is conserved among several species. A well studied example is the role of FOXO homologous DAF-16 (Caenorhabditis elegans) in aging^85^**. **Loss of FOXO3 in human skin fibroblasts results in aging specific morphological changes, suggesting that FOXO3 is necessary for maintenance or promotion of cellular longevity^86^. Moreover, loss of FOXO3 activity leads to decreased MnSOD and enhanced cell injury in vascular smooth muscle explanted from aged mice, due to limied ROS inactivation^87^. Thus, cellular lifespan maintenance requires a certain level of FOXOs activity and this is mainly achieved by inactivation of AKT activity^88^. FOXO proteins do not only play an important protective role during senescence/aging, but also during exercise.**It was previously suggested that FOXOs may at least partially be involved in the exercise-induced beneficial cardiac effects. Interestingly, exercise induces an upregulation of FOXO3 and SIRT1 in the heart, with subsequent increased MnSOD, catalase and GAD45alpha, and decreased Cyclin D2, suggesting the protective role of FOXO proteins^89^. **

Interestingly, other studies revealed that, in conditions of oxidative stress, FOXO3 is also able to induce apoptosis by triggering a Fas-mediated death pathway in cultured motoneurons, by activating TRAIL, BH3-only proteins Noxa and Bim, and by promoting pro-apoptotic activity of p53^20^. Additional reports suggest that suppression of FOXO proteins expression during oxidative stress can be protective to some extent for cells, since protein inhibition or gene knockdown of FOXO1 and FOXO3 decreases the ischemic infarct size in the brain, protects the metabotropic glutamate receptors during vascular injury, enhances pancreatic β-cell or neuronal survival through NAD + precursors and provide trophic factor protection with erythropoietin (EPO) and neurotrophins^20^. This suggests that while a certain level of FOXO3 activation is required in cellular stress resistance, sustained activation or activation over a threshold of FOXO3 is detrimental and may induce apoptosis. Thus, FOXO3 plays an important role in cellular decision during stress, helping the cells to survive by multiple mechanisms (such as DNA repair, ROS inactivation etc) and inducing apoptosis when DNA or cellular damage can’t be repaired.

### Immune response

FOXO proteins play a central role in maintaining the immune response of the human body. Cell type-specific deletions of FOXO1 and/or FOXO3 in mice revealed an important role of FOXO proteins in regulating immunological homeostasis and tolerance, by controlling the function and development of B and T lymphocytes. Thus, FOXO1 and FOXO3 are essential transcription factors involved in early B cell development and peripheral B cell function, since early deletion of FOXO1 blocked B cell differentiation at the pro-B cell stage. This is due to a defect in interleukin 7 receptor alpha (IL-7Ralpha) expression. Deletion of FOXO1 in peripheral B cells was associated with defective expression of both CD62L and AID, with subsequent failure in class-switch recombination and reduced IgG production upon immunization. Moreover, it is known that the PI3K-AKT-mediated inactivation of FOXO1 is essential for the optimal proliferation of B cells, while ectopic expression of PI3K-independent variants of FOXO1 or FOXO3 (active triple mutants at the AKT phosphorylation sites) resulted in cell cycle arrest and increased cell death in B cells^90^. Another study on mice with T cell-specific deletion of FOXO1 showed a decreased expression of IL-7 receptor on mature T-cells, suggesting that IL7-R is a transcriptional target of FOXO1, which is mediating through binding with IL-7 the survival and homeostatic proliferation of peripheral T cell^91^.

Excesive inflammatory cells may become harmful for the human body through the generation of ROS and through the production of cytokines. Studies performed on mice deficient of FOXO3 ilustrated lymphoproliferation, inflammation of the salivary glands, lung, and kidney, and increased activity of helper T cells. These observations demonstrate the beneficial role of FOXO proteins in human body, by preventing T cell hyperactivity^20^**. **Also, it was demonstrated that miR-182 has a central role in the late phase of clonal expansion of the helper T lymphocytes, by inducing IL-2 which is able to inactivate FOXO1. FOXO1 was found to be a suppressor of proliferation in resting helper T lymphocytes and, in order to allow proliferation, T cell activation via TCR/CD28 and IL-2R signaling must inhibit FOXO1^90^. Other studies reported that T cells derived from Bim and Puma deficient mice were resistant to apoptosis after IL-2 deprivation, demonstrating that FOXO3, through upregulation of Bim, Puma and p27^Kip1^ is important for the induction of cell cycle arrest and apoptosis of T cells, in the absence of cytokines^48^. FOXO proteins may be benefical for autoimmune disorders by inducing a Fas mediated apoptosis that target activated T cells, followed by a decrease in cytokine stimulation in patients with autoimmune lymphoproliferative syndromes^20^.

## 4. Implications of FOXO proteins in pathological processes and associated diseases

FOXO proteins play an important role not only during physiological cellular processes, but also in few pathologies, such as cancer. FOXOs are well known tumor suppressor proteins.

Although FOXO proteins protect the human body by playing a central role in a wide range of mainly physiological functions (as described earlier), under some circumstances, FOXO’s roles can become harmful for the human cells. This is because FOXO is also involved in a number of pathological functions, such as inflammation and muscle atrophy. For example, apoptosis places FOXO proteins on the good side when it leads to tumor suppression. However, cellular apoptosis can become itself a significant component for pathology in diseases such as neurodegenerative disease, diabetes mellitus (DM), and cardiovascular injury^40,41,92,93^.

### Cancer: FOXO proteins are tumor suppressors

FOXO proteins are inactivated in a wide variety of malignancies, eiher posttranslational (mainly by PI3K-AKT mediated phosphorlation) or by fusion mutations (such as PAX3-FOXO1; MLL-FOXO3, MLL-FOXO4)^94,95^. Inactivation of FOXO by PI3K-AKT pathway activation is a common feature of many malignancies, such as prostate cancer, breast cancer, leukemia and glioblastoma^69^**. **Conditional deletion of FOXO family members in mice leads to the lymphomas and hemangiomas, which suggests that loss of FOXOs maintains or promotes survival of tumor cells^96^.**FOXOs loss or inactivation is known to play an important role in cancer tumorigenesis or progression, *in vivo*^97^. For example, a recent study described a significant correlation between low expression of FOXO3 and a poor prognosis for gastric cancer patients, bringing evidence that FOXO3 could be a valuable prognostic biomarker for patients with gastric cancer^98^. In addition, FOXO3 overexpression was shown to reduce motility, invasiveness, and aggressiveness in estrogen receptor α-positive (ERα+) breast cancer cells^39^.

FOXO proteins exert their tumor suppressor functions predominantly by promoting cell cycle arrest, apoptosis, ROS inactivation and DNA repair, through expression of their target genes^47,99^. For instance, FOXO3-induced expression of Bim, a pro-apoptotic member of the Bcl-2 family of proteins induced a caspase-dependent cell death in several types of cancers, such as in chronic leukemias, breast and gastric cancers^27^.****Similarly, FOXO-induced TRAIL and NOXA expression induces apoptosis in many malignancies, including leukemias^100^.

Interestingly, FOXO3 can also inhibit the proto-oncogene c-myc, indirectly controlling the transcription of a wide set of target genes implicated in cell survival, cell cycle, apoptosis and tumorigenesis. Myc is a transcription factor and a well known promoter of survival, proliferation and tumorigenesis, being found upregulated in a large variety of malignancies.**FOXO3 induces the expression of Mxi1, which is a transcriptional suppressor of c-Myc, thereby inactivating Myc-dependent transcription. Mxi1 also plays a role in FOXO-induced proliferation inhibition. Similarly, FOXO-induced suppression of proliferation in colon carcinoma cell lines may involve the Mad/Mxd family of proteins, which are known Myc target genes^101^.

Noteworthy, activation of FOXO proteins not only induces cell cycle arrest or apoptosis, but also a differentiation program.**In chronic myeloid leukemia (CML), FOXO3 can induce CML leukemic cells differentiation, by inhibiting the expression of Id1 (Inhibitor of DNA binding 1). CML is characterized by the presence of the Bcr-Abl fusion protein, which is a constitutive active kinase, controlling many crucial downstream pathways implicated in cell cycle, proliferation, apoptosis and cell adhesion. Bcr-Abl activates PI3K-AKT pathway, inactivating FOXO proteins and their pro-apopotic and cell cycle arrest signals^27^**. **The use of the Tyrosine Kinase Inhibitors (TKIs) and of the proteasome inhibitor Bortezomib results in inhibition of Bcr-Abl activity and its downstream pathways, with a subsequent activation of FOXO proteins^102^. TKIs treatment induces a FOXO-dependent suppression of Id1 expression, leading to K562 Bcr-Abl positive cell line differentiation. Thus, Bcr-Abl can maintain the leukemic state not only by promoting proliferation, cell cycle progression and inhibiting apoptosis, but by also inhibiting FOXO-mediated differentiation^27^.

### Inflammation (rheumatoid arthritis, osteoarthritis, systemic lupus erythematosus)

It was shown that loss of functional FOXO proteins lead to**inflammatory cell activation in several disorders, with the subsequent cellular damage, through oxidative stress and excess of cytokines. Inactivation of FOXO3 in T lymphocytes, as well as inactivation of FOXO1 and FOXO4 in synovial macrophages in patients with **rheumatoid arthritis** and *osteoarthritis* result in inflammatory cell activation. In addition, loss of FOXO proteins may be a potential etiology for **systemic lupus erythematosus (SLE)** and *rheumatoid arthritis*, since FOXO1 gene transcript levels are downregulated in peripheral blood mononuclear cells**of these patients^20^*****. *****A link between inflammation, insulin resistance and FOXO transcription factors was previously suggested when downregulated FOXO1 decreased the levels of C/EBP beta transcription factor in adipocytes, with the subsequent reduction in expression of the pro-inflammatory cytokines CCL2 (chemokine ligand 2) and IL-6^103^. Thus, FOXO1 indirectly might induce an inflammatory status of the adipose tissue, responsible for the insulin resistance in type 2 diabetes. Yet, there are studies that describe the ability of FOXO proteins to directly induce inflammation through upregulation of the inflammatory cytokine IL-1b^104^. However, further experiments are required in order to clearly understand the context related mechanisms of FOXO-dependent modulation of inflammation.

### Muscle atrophy by proteolysis

Muscle atrophy appears in a variety of diseases, including cancer, diabetes and sepsis, and is characterized by accelerated proteolysis that can be induced through two pathways: the ubiquitin – proteasome pathway and through lysosomal pathway, as a consequence of autophagy^27^. Studies revealed that FOXO proteins can induce muscle atrophy, characterized by decreased muscle function^105^. Thus, FOXO proteins have been shown to increase the transcription of key regulators of both lysosomal and proteasomal proteolysis: the autophagy followed by the lysosomal proteolysis is stimulated by upregulation of Bnip3, LC3, and Gabarapl1, while the proteasomal proteolysis is induced by increasing the ubiquitin ligase atrogin1. It was also shown that FOXO3 activity is both required and sufficient for induction of autophagy in muscle cells, since studies performed on adult muscle fibers from mice show that the ectopic expression of an active FOXO3 mutant leads to lysosomal proteolysis after formation of autophagosomes, while the knockdown of FOXO3 in these muscles fibers blocked autophagosome formation after starvation^106,107^.

Autophagy has a critical role in maintaining the cellular and metabolic homeostasis. It seems that the metabolic status of the cell strongly influences this process in both normal cells and cancer cells, despite the profound differences in their metabolism^108^. In cancer cells, ATP is predominantly produced through the constitutive activation of aerobic glycolysis, process that is modulated by the transcription factor HIF1α^108^**. **Since p38α is required to maintain the levels of HIF1α target genes, researchers demonstrated that in colorectal cancer cells, the inhibition of p38α causes a rapid drop in ATP levels, with an acute energy need which activates FOXO3 in an AMPK-dependent manner, in order to induce autophagy, cell cycle arrest and cell death in these cells^96,108^.

Moreover, the knockdown of FOXO3 was sufficient to induce hypertrophy in cultured neonatal rat cardiomyocytes. In these cells, stimulation with insulin inhibits FOXO3 function, with subsequent downregulation of the antioxidant enzyme catalase and increased levels of ROS, that in low levels may act as second messengers for intracellular signaling, making possible the increase in cell size^109^. Atrogin 1, upregulated by FOXO1 and FOXO3, plays important roles in cardiovascular system, since mice lacking atrogin-1 are susceptible to cardiac hypertrophy^20^. FOXO proteins are inducing atrophy of differentiated cardiac and skeletal muscle cells through protein synthesis inhibition, which leads to a decrease in cell size^69^. In skeletal muscle, this mechanism involves myostatin, a FOXO transcriptional target and a secreted molecule that can induce atrophy by protein synthesis inhibition^110^.

### Other pathological processes: metabolic, cardiovascular and reproductive disorders

FOXO1 might be implicated in the development or progression of *type 2 diabetes*, since increased FOXO1 expression in diabetic mouse liver is associated with increased expression of PEPCK and G6Pase, and inhibition of FOXO1 activity downregulated both PEPCK and G6Pase expression and normalized the blood glucose levels. Thus, FOXO1-mediated expression of G6Pase and PEPCK is critical for gluconeogenesis in the liver during fasting, but its deregulation may be involved in diabetes etiology^111^. Physiological functions of FOXO take place under certain circumstances, since sometimes the ability to maintain the proper control is overwhelmed^20^. Experiments on insulin-producing mouse pancreatic beta cells (betaTC-6) show that chronic exposure to high glucose activates FOXO transcription factors and leads to upregulation of endogenous inflammatory cytokines interleukin-1beta (IL-1beta) and suppressors of cytokine signalling (SOCS). These events trigger the activation of caspase-3 with subsequent apoptosis, suggesting a new mechanism that leads to the destruction of endocrine pancreas in type 2 diabetes^92^. Also, exposure to high glucose of the cardiac microvascular endothelial cells (CMECs) isolated from hearts of adult rats show that FOXO transcription factors leads to reactive oxygen species (ROS) accumulation and apoptosis, suggesting that FOXOs might be involved in microvascular complications of diabetes^93^. FOXO is also involved in insulin resistance and metabolic syndrome, since the activation of FOXO1 in cardiomyocytes leads to increased AKT activity and attenuated cellular response to insulin, followed by decreased glucose uptake^112^.

Interestingly, clinical studies regarding metabolic status profile on age-related diseases, fertility, fecundity and mortality revealed higher HbA1c levels and increased mortality risk associated with specific haplotypes of FOXO1^113^.

FOXO proteins are also activated in an attempt to protect the human body against the oxidative stress resulted due to hyperglycemia that leads to increased production of ROS in endothelial cells, liver cells, and pancreatic β-cells. This hyperglycemia-dependent ROS increase leads to a subsequent development of insulin resistance and significant neurodegenerative and cardiovascular diseases in the patients with diabetus mellitus^20^.

FOXOs are necessary for endothelial cell development and *angiogenesis*, since mice that are deficient in FOXO1 lack development of the vascular system and die by embryonic day eleven^114^. In addition, FOXO1 and FOXO3 were shown to be the most abundant FOXO isoforms in mature endothelial cells, having also an important role in the regulation of postnatal vessel formation, not only in the embryogenesis^36^. Unfortunately, angiogenesis is not only involved in critical physiological processes, such as embryogenesis and postnatal vessel formation, but it is also involved in pathological events, such as chronic inflammation and tumor growth^115^. Thus, it is fascinating how the angiogenesis mediated by FOXO proteins may become a negative element for the organism, antagonizing the tumor suppresor’s main function through new vessel formation, that can lead to tumor cell growth^116^.**

FOXO3 was associated with both cardiomyocyte survival after oxidative stress and *heart muscle loss* with subsequent *ventricular dysfunction*^43,117^. FOXOs are activated after AKT inhibition by insulin or other factors, leading to Atrogin-1 induction, which results in a suppression of heart muscle cell size^43^.

FOXO3 proteins seem to inhibit the vascular smooth muscle cell proliferation and growth in a rat balloon carotid arterial injury model, suggesting a role of FOXOs in the regulation of vascular tone and systemic arterial blood pressure, preventing or at least lessening the effects of **atherosclerosis and hypertension^118^**. Also, decreased FOXO1 expression due to high flow states in vessels leads to proliferation of vascular smooth muscle cells, vascular neointimal hyperplasia, and subsequent hypertension^45^. Moreover, experiments on low-density lipoprotein (Ldl) receptor knockout mice resulted in the prevention of atherosclerosis when the triple ablation of FOXO1, FOXO3 and FOXO4 was induced in endothelial cells^44^. Interestingly, the same experiment on myeloid cells lead to more severe atherosclerosis compared to the controls, explained by authors through increased proliferation of granulocyte-monocyte progenitors and high levels of inducible nitric oxide synthase (iNOS) and oxidative stress, which predispose to atherosclerosis^119^.

Noteworthy, analysis of mouse oocytes revealed overexpressed FOXO3 transcription factors in primordial and early primary follicles, but downregulated FOXO in primary and more developed follicles, suggesting that FOXO proteins also have reproductive functions, modulating oocyte and follicular cell maturation. To confirm the hypothesis, constitutively active FOXO3 was induced in transgenic mouse oocytes in primary and more developed follicles, which affected the oocyte growth and follicular development, leading to *anovulation *and *luteinization of unruptured follicles*, with subsequent *infertility^46^*. In addition, FOXO3 and FOXO1 mutations were detected in a small percentage of womens with **premature ovarian failure^120^.**

## 5. Critical functions of FOXO transcription factors in stem cells and their role in cancer stem cells

Involvement of FOXO family of transcription factors in stem cells self-renewal, survival, proliferation and differentiation is currently under investigation. FOXOs have been shown to play critical functions in maintaining self-renewal potential and quiescence of hematopoietic stem cells, however, the mechanisms of these processes are not yet well understood^1,69^. Recent reports show that FOXO-mediated regulation of cell cycle, oxidative stress and apoptosis plays an important role in these processes^69^.

Stem cells are characterized by the capacity of self-renewing and the ability to differentiate^121^. They are necessary in maintenance and propagation of several adult tissues, including but not limited to blood, skin and gastro-intestinal epithelium.**Adult stem cells or cells with stem cell properties were also found in other critical organs/systems, such as the central nervous system and the lung^69^.

Previous studies suggested that hematopoietic stem cells are sensitive to reactive oxygen species (ROS) levels. FOXO family members are known to play a central role in ROS detection and in inducing an adaptative response after ROS exposure, by inducing the expression of critical enzymes that neutralise ROS, such as catalase and manganase superoxide dismutase (MnSOD). Interestingly, FOXO1, FOXO3, and FOXO4 inactivation leads to an upregulation of ROS levels in hematopoietic stem cells and their death^121^**. **Deletion of these three FOXO family members in mice revealed their importance in controlling ROS in stem cells, *in vivo*^27^. While the number of hematopoietic stem cells in bone marrow of FOXO-deficient mice is low, an increase in myeloid progenitor cells in blood is observed. Moreover, the repopulation ability is decreased in the absence of FOXOs and the treatment of FOXO-deficient mice with N-Acetylcysteine (NAC) at least partially rescued these effects^27^.**

Thus, persistent AKT activation, which induces inactivation of FOXO transcription factors by AKT-mediated phosphorylation, results in ROS-induced cell death. This is due to the fact that the cells can’t synthesize the FOXO-dependent ROS neutralizing factors catalase and MnSOD^121^**. **Between FOXO family members, the most important regulator of hematopoietc stem cells survival and self-renewal is FOXO3, since FOXO3 knockdown induces hematopoietic stem cells depletion^121^. ROS neutralizing agent N-Acetylcysteine (NAC) can rescue hematopoietic stem cells quiescence and at least partially resque their ROS-induced loss^121^. All these results suggest that FOXO1, FOXO3 and FOXO4-induced resistance to ROS is critical in maintaining homeostasis of hematopoietic stem cells in bone marrow^27^.

PTEN tumor suppressor was revealed as an important modulator of hematopoietic stem cells self-renewal and survival. PTEN is a major inhibitor of AKT activation. Depletion of PTEN, induces AKT activation and subsequent FOXO inactivation. It is likely that FOXOs mediate at least a part of the PTEN effects on hematopoietic stem cells^121^.

Although FOXO3 was established as the most important FOXO family member regulator of hematopoietic stem cell’s self-renewal, FOXO1 is critical for the human embryonic stem cells (hESC) pluripotency maintenance. FOXO1-induced SOX2 and OCT4 expression is one of the mechanisms responsible for this process. Interestingly, in embryonic stem cells AKT is not the major regulator of FOXO1^71^.

Fascinating, a recent study brings evidence that FOXO is a critical regulator of stem cell maintenance in *Hydra vulgaris*, a member of the phylogenetically old animal phyla *Cnidaria,* which has been suggested to be biologically immortal due to the unlimited self-renewal capacity of their stem cells^38,122^.

FOXO transcription factors are not only required for maintenance of somatic stem cells, but they also play an important role in cancer stem cells^71^. Recent reports provided evidence that in many types of malignancies, such as leukemia, colon, or gastro-intestinal malignanciens, a small population of cells similar to stem cells exists^121^. These cells, called cancer stem cells, have the potential of forming new tumors. Notably, most of the time, the cancer stem cells are resistant to current cancer treatments, and these therapies may result in cancer stem cells enrichment. These cells serve as a starting point in cancer recurrence^121^. Similarities between stem cells and cancer stem cells were best described in the hematopoietic system, where similar surface markes and signal transduction patterns were described between the hematopoietic stem cells and leukemia-initiating cells^121^.

Presented results have not only implications in uncovering the stem cells/cancer stem cells regulation, self-renewal and differentiation, but also for the development of novel therapeutic strategies in cancer, degenerative diseases and many other pathologies^71^.

## 6. Expression of FOXO family members in various tissues

FOXO family members are expressed in almost every tissue of the human body,**including**the nervous system, cardiovascular system, reproductive system of males and females, lung, liver, spleen, pancreas, thymus, and skeletal muscle. However, each member of the FOXO family has its own expression pattern, since they are not equally expressed in all tissues^20^. FOXO1 is better represented in adipose tissue^27^. FOXO3 has the highest expression in liver, but it is also being predominantly expressed in heart, brain, kidneys, and ovaries, while FOXO4 is found mainly in the muscle and heart^20,27^. The newest member of the transcription family, FOXO6, is present in the brain. The association of this member with other tissues is still a matter of study^123^.

Cell lines (immortalized and/or cancer cells) are some of the most used and useful tools for studying the structure, function, regulation and expression of proteins in general, and FOXO family members in particular. FOXO members expression in different cell lines (including NCI 60 group of cell lines) is partially known^124,125^. FOXO1 is found to be expressed at high levels in IGROV 1 (human ovarian carcinoma cells), astrocyte cells, RL 7 (human follicular lymphoma cells), HT29 (human colon carcinoma cells) and HEK 293 (human embryonic kidney cells). FOXO3 high levels were especially found in SHSYSY (human neuroblastoma cell line), RPMI 8226 (human myeloma cells), MCF 7 (human breast adenocarcinoma cell line), LNCAP (human prostate adenocarcinoma cells), U251 (human glioma cells). FOXO4 is mostly expressed in CCRT CEM (human leukemia cell line), ALVA 31 (human prostate tumor cells), HL 60 (human promyelocytic leukemia cell line), TK 10 (human renal adenocarcinoma cells), MALME 3M (human malignant melanoma cell line) and HEP G2 (human hepatocellular carcinoma cells)^126-128^.

Knowing the qualitative and quantitative expression pattern of FOXO family members is important in elucidating their functions and regulation. Thus, databases summarizing these patterns in various tissues and cell lines are very helpful and necessary. For example, BioGPS presents experimental results showing the mRNA expression levels of a wide number of genes in most of the human tissues and many (mostly human) cell lines^126-128^.

Regulation of FOXOs expression is not yet well understood. P300 was shown to control FOXO1 gene expression by binding to the proximal region of the FOXO1 promoter (CRE tandem sites)^129^. Non-coding RNAs were shown to suppress translation of FOXO family members in various tissues and contexts. In particular, the microRNAs-dependent inhibition of FOXO1 and FOXO3 expression is better studied and is summariez below, in chapter 7.

However, the main transcription factors implicated in FOXOs expression are not well understood. Interestingly, methylation of FOX promotors induces suppression of their expression. As an example, promoter methylation induced by BRAF results in inactivation of FOX genes expression^130^.

## 7. FOXO and non-coding RNAs

The miRNAs are 18–28 nucleotide-long noncoding RNA molecules with an important role in post-transcrip­tional regulation of protein expression that regulate a variety of cellular processes, including cell differentiation, cell cycle progression and apoptosis. These miRNAs can function as oncogenes or tumor suppressors, and oncogenic miRNAs (oncomiRs) are upregulated in cancer cells. In cancer, miRNAs were found to be situated both upstream and downstream of the carcinogenesis process and modified expression of some miRNAs is the outcome of carcinogenic transformation or progression, revealing that miRNAs may be potential diag­nostic or prognostic tools in cancer^131^. For instance, microRNAsgained a special attention in melanoma studies and the altered pattern of miRNA in melanoma seems to be related to apoptosis (miR-15b), cell cycle (miR-193b) and invasion/metastasis (miR-182)^132^.

### FOXOs regulation by microRNAs

The microRNA (miRNA)-mediated regulation of FOXO transcription factors was demonstrated by several groups within the last three years. miR-182 has been shown to specifically target FOXO transcription factors irrespective of cell type, since miR-182 seems to target FOXO3 in melanoma cells, FOXO1 in breast cancer cells and FOXO1 in activated helper T (Th) lymphocytes^90^. Thus, in melanoma cells, miR-182 modulate the expression of both FOXO3 and microphthalmia-associated transcription factor (MITF). The inhibition of miR-182 by anti-miRs (blocking antisense oligonucleotides) hindered melanoma cell migration and triggered their apoptosis^90^. In breast cancer cells, FOXO1 was coordinately targeted by miR-27a, miR-96, and miR-182, while the inhibition of each miRNA resulted in induced levels of FOXO1 and reduced breast cancer cell survival^90^. miR-182 also targets FOXO1 in osteoblasts lineage cells in order to inhibit osteoblast proliferation and differentiation, repressing the osteogenesis^133^.

Many other microRNAs were shown to regulate FOXOs activity and functions, including apoptosis or cell cycle. MiR-96 has been shown to regulate FOXO1-induced apoptosis in transitional cell carcinoma^134^. Also, increased expression of miR-96 in breast cancer cells have been shown to downregulate FOXO3 transcription factor with consequent induction of cell proliferation^135^. Also, increased miR-96, miR-182 and miR-183 downregulate the expression of FOXO1 transcription factor in classical Hodgkin lymphoma (cHL) cell lines, suggesting that decreased FOXO1 expression is involved in lymphomagenesis^136^. Moreover, a recent study performed on DU145 and LNCaP human prostate cancer cells show that upregulated microRNA-370 induces proliferation due to downregulation of the FOXO1 transcription factor^137^. In addition, miR-155, which is highly induced in mature activated T and B cells and in T_reg_ cells, has recently been shown to target FOXO3 in T cells, but it remains to be shown whether FOXO3 via miR-155 contributes to the observed phenotypes in B and T lymphocytes^90^.

New strategies to modulate FOXO expression may now be developed since the discovery of miRNAs targeting FOXO transcription factors^90^. However, some difficulties may appear in the miRNA-based therapeutic manipulation of FOXO transcription factors. For example, a single miRNA may target hundreds of genes among FOXOs, and the therapeutic manipulation of a specific miRNA could have unanticipated adverse effects by influencing whole gene networks, while having only moderate effects on FOXO genes^90^**. **Since delivery of miR-182 mimics could induce lymphoproliferative disease or other forms of cancer by systemic repression of FOXO transcription factors, another challenge in the miRNA-FOXO therapeutic manipulation is the specific delivery of the miRNA into the target cell,**in order to avoid adverse effects in other cell types or tissues^90^.

### microRNAs functions mediated by FOXOs

Several microRNAs were identified as being targets of FOXO transcription factors. An AKT - FOXO - miR-30d signaling pathway was recently identified. After inhibition of AKT, activated FOXO3 leads to upregulation of miR-30d. miR-30d acts as a tumor suppressor in renal cell carcinoma, further inhibiting the oncoprotein metadherin (MTDH)^138^.

Another recent study shows that FOXO1 stimulates the expression of a microRNA cluster located on a X chromosome, in a direct manner, dependent on RNA polymerase II, but not on the de novo protein synthesis. Thus, FOXO1 upregulates miR-506, miR-508, miR-513c, miR-513a-1, miR-513a-2. Also, the same study shows that inhibition of PI3K-AKT axis in LNCaP and MCF7 human carcinoma cell lines is followed by increased miR-506. As suggested by authors, miRNAs could be valuable biomarkers of FOXO activity^139^.

A study performed on primary cultures of neural stem/progenitor cells (NSPCs) from adult mice show that the expression levels of the miR-106b~25 cluster members (miR-106b, miR-93, and miR-25) is modulated by FOXO3 transcription factors in a complex manner^140^. The precursors of the miR-106b~25 cluster members are located on Mcm7 gene. FOXO3 directly binds to the first intron of this gene, modulating the expression of the microRNAs. Thus, FOXO3 transcription factors could be an important tool in preventing the loss of neurogenesis during aging^140^. Further studies are needed to completely understand the regulation of microRNAs expression by activated FOXO proteins.

## 8. Conclusions and future perspectives

FOXO transcription factors and tumor suppressors are ubiquitously expressed in the human body, with some specific differences between its members. As resumed above, FOXO proteins are characterized by a remarkable functional diversity, being implicated in regulation of many critical cellular functions, such as cell cycle arrest, apoptosis, oxidative detoxification, DNA damage repair, stem cell maintenance, cell differentiation, cell metabolism, angiogenesis, cardiac development, aging and others.

FOXO proteins play an important role not only during physiological cellular processes, but also in few pathologies, such as cancer. They are well known tumor suppressors proteins. Although FOXO proteins protect the human body by playing a central role in a wide range of mainly physiological functions, under some circumstances, FOXO’s roles can become harmful for the human cells. This is because FOXO is also involved in a number of pathological functions, such as inflammation, muscle atropy, and a number of physiological functions that become harmful for the organism. For example, apoptosis places FOXO proteins on the good side when it leads to tumor suppression, but in some cases cellular apoptosis can become itself a significant component for pathology in diseases such as neurodegenerative disease, diabetes mellitus (DM), and cardiovascular injury.

Interestingly, while excessive FOXO levels induce cell cycle arrest and cell death, complete knock-down of FOXOs leads to cell cycle progression impairment, suggesting that certain levels of FOXO activation are required for cell cycle progression. Moreover, FOXO proteins play a variety of roles in the cells dependending on the context.

In addition, FOXO have critical implications in both normal and cancer stem cell biology. FOXO proteins are required for adult stem cells maintenance. Novel strategies to modulate FOXO expression and activity may now be developed since the discovery of novel FOXO regulators and non-coding RNAs (such as microRNAs) targeting FOXO transcription factors.

Thus, FOXO family of transcription factors and tumor suppressors are crucial regulators of cell fate and of critical normal or pathological cell decisions. A better understanding of their structure, critical functions and regulation may contribute to the development of novel therapies for cancer and other FOXO-related diseases.

- ****FOXO proteins are characterized by a remarkable functional diversity, controlling many critical cellular processes:**** cycle arrest, apoptosis, oxidative detoxification, DNA damage repair, stem cell maintenance, cell differentiation, cell metabolism, angiogenesis, cardiac development, fertility, immune response, neuronal survival,aging and others.
